# (2-Hy­droxy-7-meth­oxy­naphthalen-1-yl)(4-methyl­phen­yl)methanone

**DOI:** 10.1107/S1600536810040614

**Published:** 2010-10-20

**Authors:** Atsushi Nagasawa, Ryosuke Mitsui, Akiko Okamoto, Noriyuki Yonezawa

**Affiliations:** a Department of Organic and Polymer Materials Chemistry, Tokyo University of Agriculture & Technology, 2-24-16 Naka-machi, Koganei, Tokyo 184-8588, Japan

## Abstract

In the title compound, C_19_H_16_O_3_, an intra­molecular O—H⋯O=C hydrogen bond is formed between the hy­droxy and carbonyl groups on the naphthalene ring system, resulting in an *S*(6) ring. The angles between the C=O bond vector and the least-squares planes of the naphthalene ring system and the benzene ring are 27.63 (6) and 47.99 (7)°, respectively. The dihedral angle between the latter planes is 61.39 (5)°. In the crystal, two mol­ecules are connected by pairs of inter­molecular O—H⋯O=C hydrogen bonds, forming centrosymmetric dimers with an *R*_2_^2^(4) graph-set motif. The mol­ecular packing features C—H⋯π interactions.

## Related literature

For electrophilic aromatic substitution of naphthalene deriva­­tives, see: Okamoto & Yonezawa (2009 ▶). For the structures of closely related compounds, see: Mitsui *et al.* (2008 ▶); Nagasawa *et al.* (2010*a* ▶,*b* ▶,*c* ▶). For hydrogen-bond motifs, see: Bernstein *et al.* (1995 ▶); Etter *et al.* (1990 ▶).
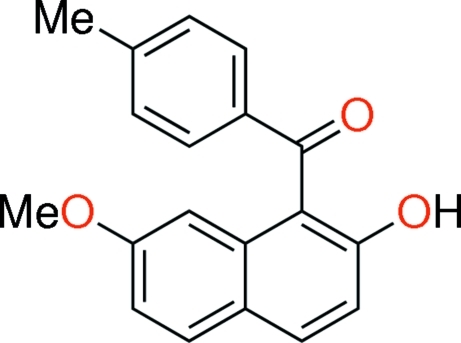


## Experimental

### 

#### Crystal data

C_19_H_16_O_3_
*M* *_r_* = 292.32
Monoclinic, 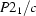

*a* = 11.1599 (2) Å
*b* = 6.05387 (11) Å
*c* = 22.0153 (4) Å
β = 90.317 (1)°
*V* = 1487.35 (5) Å^3^
*Z* = 4
Cu *K*α radiation
μ = 0.71 mm^−1^
*T* = 193 K
0.60 × 0.50 × 0.30 mm

#### Data collection

Rigaku R-AXIS RAPID diffractometer
Absorption correction: numerical (*NUMABS*; Higashi, 1999 ▶) *T*_min_ = 0.676, *T*_max_ = 0.816
25155 measured reflections
2729 independent reflections
2493 reflections with *I* > 2σ(*I*)
*R*_int_ = 0.029

#### Refinement

*R*[*F* ^2^ > 2σ(*F* ^2^)] = 0.038
*wR*(*F* ^2^) = 0.106
*S* = 1.04
2729 reflections
204 parameters
1 restraint
H atoms treated by a mixture of independent and constrained refinement
Δρ_max_ = 0.28 e Å^−3^
Δρ_min_ = −0.15 e Å^−3^

### 

Data collection: *PROCESS-AUTO* (Rigaku, 1998 ▶); cell refinement: *PROCESS-AUTO*; data reduction: *CrystalStructure* (Rigaku/MSC, 2004 ▶); program(s) used to solve structure: *SIR2004* (Burla *et al.*, 2005 ▶); program(s) used to refine structure: *SHELXL97* (Sheldrick, 2008 ▶); molecular graphics: *ORTEPIII* (Burnett & Johnson, 1996 ▶), *ORTEP* -3 for Windows (Farrugia, 1997 ▶) and *PLATON* (Spek, 2009 ▶); software used to prepare material for publication: *SHELXL97*.

## Supplementary Material

Crystal structure: contains datablocks I, global. DOI: 10.1107/S1600536810040614/dn2608sup1.cif

Structure factors: contains datablocks I. DOI: 10.1107/S1600536810040614/dn2608Isup2.hkl

Additional supplementary materials:  crystallographic information; 3D view; checkCIF report

## Figures and Tables

**Table 1:** Hydrogen-bond geometry (Å, °) CT1 and CT2 are the centroids of the C5–C10 and C12–C17 rings, respectively.

*D*—H⋯*A*	*D*—H	H⋯*A*	*D*⋯*A*	*D*—H⋯*A*
O1—H1⋯O3	0.95 (2)	1.70 (2)	2.5618 (14)	148 (2)
O1—H1⋯O3^i^	0.95 (2)	2.33 (2)	3.0083 (16)	128 (1)
C6—H6⋯CT1^ii^	0.95	2.71	3.5203 (13)	144
C17—H17⋯CT1^iii^	0.95	2.76	3.5492 (12)	141
C19—H19*C*⋯CT2^iv^	0.98	2.88	3.7834 (16)	154

Symmetry codes: (i) 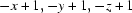; (ii) 
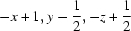
; (iii) 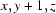; (iv) 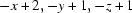.

**Fig. 1.**
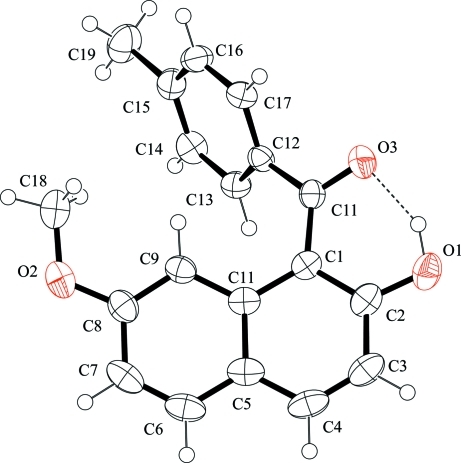
The asymmetric unit of compound (I) with the atom labeling scheme. Displacement ellipsoids are drawn at the 50% probability. H atoms are represented as small spheres of arbitrary radii. Intramolecular hydrogen bond is shown as a dashed line.

**Fig. 2.**
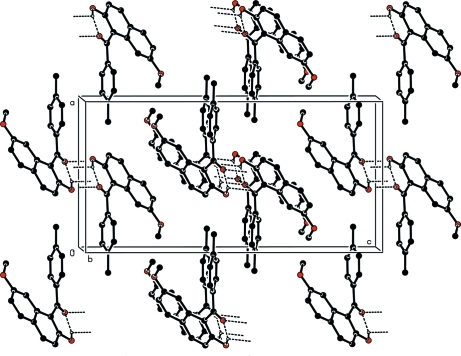
A crystal packing diagram of compound (I), viewed down the b axis. Intermolecular hydrogen bonds are shown as dashed lines. H atoms not involved in hydrogen bondings have been omitted for clarity.

## supplementary crystallographic information

### Comment

In the course of our study on selective *peri*-aroylation of 2,7-dimethoxynaphthalene (Okamoto & Yonezawa, 2009), the crystal structures of several 1-monoaroylated naphthalene compounds have been clarified along with 1,8-diaroylnaphthalene. Furthermore, selective demethylation reaction of the 1-monoaroylated 2,7-dimethoxynaphthalene compounds has been proved to yield the 1-monoaroyl-2-hydroxynaphthalene compounds, which are rather susceptible to imination reaction. In this course, we recently reported crystal structure of several imine compounds prepared from 1-monoaroylated naphthalene derivatives having 2-hydroxy group exemplified by 1-[(4-chlorophenyl)(phenylimino)methyl]-7-methoxy-2-naphthol-1,4- diazabicyclo[2.2.2]octane (2/1) (Nagasawa *et al.*, 2010*a*) and 1-[phenyl(3-nitrophenylimino)methyl]-7-methoxy-2-naphthol (Nagasawa *et al.*, 2010*c*) derived from (4-chlorophenyl)(2-hydroxy-7-methoxynaphthalen-1-yl)methanone (Mitsui *et al*., 2008) and (2-hydroxy-7-methoxynaphthalen-1-yl)(phenyl)methanone (Nagasawa *et al.*, 2010*b*), respectively. As a part of our ongoing studies on the synthesis and crystal structure analysis of aroylated naphthalene homologues, we prepared and analysed the crystal structure of the title compound (I).

In the molecule (I), the intramolecular O—H···O═C hydrogen bond that forms a six-membered S(6) ring (Etter *et al.*, 1990; Bernstein *et al.*, 1995) including carbonyl and hydroxy groups on the naphthalene ring (Table 1, Fig. 1). The conformation of these groups resembles to that of (2-hydroxy-7-methoxynaphthalen-1-yl)(phenyl)methanone (Nagasawa *et al.*, 2010*b*). The angles of C═O bond vector against the least-squares plane of the naphthalene ring (C1–C10) and benzene ring (C12–C17) are 27.63 (6) and 47.99 (7)°, respectively. The dihedral angle between the naphthalene ring (C1–C10) and benzene ring (C12–C17) is 61.39 (5)°.

In the crystal structure, O—H···O═C intermolecular hydrogen bonds between the hydroxy group and the carbonyl one on the naphthalene ring (Table 1) form a centrosymmetric dimer (Fig. 2) with a R_2_^2^(4) graph set motif (Etter *et al.*, 1990; Bernstein *et al.*, 1995). The molecular packing of (I) is mainly stabilized by intermolecular C—H···π hydrogen bond involving the centroid CT1 of the C5—C10 ring and the centroid CT2 of the C12–C17 phenyl ring. (Table 1).

### Experimental

To a solution of 1-(4-methylbenzoyl)-2,7-dimethoxynaphthalene (3.07 g, 10 mmol) in CH_2_Cl_2_ (100 ml) was added AlCl_3_ (6.65 g, 50 mmol). The reaction mixture was refluxed for 30 min giving a dark red solution, which was then poured into H_2_O (30 ml). The aqueous layer was extracted with CHCl_3 _(30 ml × 3). The combined organic layers were washed with brine (30 ml × 3) and dried over MgSO_4_ overnight. The solvent was removed *in vacuo* and the crude material was purified by recrystallization from hexane to give compound (I) as yellow platelets (m.p. 385.5–386.00 K, yield 1.76 g, 60%).

Spectroscopic Data: ^1^H NMR (300 MHz, CDCl_3_) δ 11.40 (s, 1H), 7.83 (d, *J* = 8.9 Hz, 1H), 7.61 (d, *J* = 8.6 Hz, 1H), 7.52 (d, *J* = 7.9 Hz, 2H), 7.22 (d, *J* = 7.9 Hz, 2H), 7.07 (d, *J* = 8.9 Hz, 1H), 6.89 (dd, *J* = 8.9, 2.4 Hz, 1H), 6.64 (d, *J* = 2.4 Hz, 1H), 3.31 (s, 3H), 2.41 (s, 3H); ^13^C NMR (75 MHz, CDCl_3_) δ 200.0, 162.3, 158.1, 143.2, 138.0, 136.1, 134.2, 130.0, 129.5, 129.3, 123.8, 116.5, 115.8, 114.0, 106.6, 54.5, 21.7; IR (KBr): 3443, 2929, 1620, 1561, 1514, 1233; HRMS (*m/z*): [*M* + H]^+^ calcd for C_19_H_17_O_3_, 293.1178; found, 293.1189.

### Refinement

All the H-atoms could be located in difference Fourier maps. The coordinates of the OH hydrogen atom were refined using restraint (O1—H1 = 0.95 (2) Å) with *U*_iso_(H) = 1.5*U*_eq_(O). The H atoms attached to carbon were introduced in calculated positions and treated as riding on their parent atoms with C—H= 0.98 Å (methyl) or 0.95 Å (aromatic) with *U*_iso_(H) = 1.2*U*_eq_(C_aromatic_) or *U*_iso_(H) = 1.5*U*_eq_(C_methyl_).

### Crystal data

**Table:**

C_19_H_16_O_3_	*F*(000) = 616
*M**_r_* = 292.32	*D*_x_ = 1.305 Mg m^−^^3^
Monoclinic, *P*2_1_/*c*	Melting point = 385.5–386.0 K
Hall symbol: -P 2ybc	Cu *K*α radiation, λ = 1.54187 Å
*a* = 11.1599 (2) Å	Cell parameters from 21159 reflections
*b* = 6.05387 (11) Å	θ = 4.0–68.2°
*c* = 22.0153 (4) Å	µ = 0.71 mm^−^^1^
β = 90.317 (1)°	*T* = 193 K
*V* = 1487.35 (5) Å^3^	Block, colorless
*Z* = 4	0.60 × 0.50 × 0.30 mm

### Data collection

**Table:**

Rigaku R-AXIS RAPID diffractometer	2729 independent reflections
Radiation source: rotating anode	2493 reflections with *I* > 2σ(*I*)
graphite	*R*_int_ = 0.029
Detector resolution: 10.00 pixels mm^-1^	θ_max_ = 68.2°, θ_min_ = 4.0°
ω scans	*h* = −13→13
Absorption correction: numerical (*NUMABS*; Higashi, 1999)	*k* = −7→7
*T*_min_ = 0.676, *T*_max_ = 0.816	*l* = −26→26
25155 measured reflections	

### Refinement

**Table:**

Refinement on *F*^2^	Primary atom site location: structure-invariant direct methods
Least-squares matrix: full	Secondary atom site location: difference Fourier map
*R*[*F*^2^ > 2σ(*F*^2^)] = 0.038	Hydrogen site location: inferred from neighbouring sites
*wR*(*F*^2^) = 0.106	H atoms treated by a mixture of independent and constrained refinement
*S* = 1.03	*w* = 1/[σ^2^(*F*_o_^2^) + (0.0563*P*)^2^ + 0.4274*P*] where *P* = (*F*_o_^2^ + 2*F*_c_^2^)/3
2729 reflections	(Δ/σ)_max_ < 0.001
204 parameters	Δρ_max_ = 0.28 e Å^−^^3^
1 restraint	Δρ_min_ = −0.15 e Å^−^^3^

### Special details

**Table:**

Geometry. All esds (except the esd in the dihedral angle between two l.s. planes) are estimated using the full covariance matrix. The cell esds are taken into account individually in the estimation of esds in distances, angles and torsion angles; correlations between esds in cell parameters are only used when they are defined by crystal symmetry. An approximate (isotropic) treatment of cell esds is used for estimating esds involving l.s. planes.
Refinement. Refinement of *F*^2^ against ALL reflections. The weighted *R*-factor *wR* and goodness of fit *S* are based on *F*^2^, conventional *R*-factors *R* are based on *F*, with *F* set to zero for negative *F*^2^. The threshold expression of *F*^2^ > σ(*F*^2^) is used only for calculating *R*-factors(gt) *etc*. and is not relevant to the choice of reflections for refinement. *R*-factors based on *F*^2^ are statistically about twice as large as those based on *F*, and *R*- factors based on ALL data will be even larger.

### Fractional atomic coordinates and isotropic or equivalent isotropic displacement parameters (Å^2^)

**Table:**

	*x*	*y*	*z*	*U*_iso_*/*U*_eq_	
O1	0.39263 (8)	0.16739 (19)	0.46840 (5)	0.0479 (3)	
H1	0.4401 (16)	0.298 (3)	0.4719 (9)	0.072*	
O2	0.83634 (10)	−0.07553 (19)	0.23277 (5)	0.0539 (3)	
O3	0.56764 (8)	0.43447 (17)	0.45225 (4)	0.0464 (3)	
C1	0.55371 (10)	0.1035 (2)	0.39700 (5)	0.0324 (3)	
C2	0.44354 (11)	0.0500 (2)	0.42366 (6)	0.0391 (3)	
C3	0.38007 (12)	−0.1414 (3)	0.40634 (7)	0.0476 (4)	
H3	0.3062	−0.1761	0.4254	0.057*	
C4	0.42384 (12)	−0.2755 (3)	0.36282 (7)	0.0473 (4)	
H4	0.3814	−0.4065	0.3527	0.057*	
C5	0.53176 (12)	−0.2259 (2)	0.33185 (6)	0.0390 (3)	
C6	0.57478 (13)	−0.3633 (2)	0.28499 (6)	0.0456 (4)	
H6	0.5324	−0.4947	0.2752	0.055*	
C7	0.67500 (14)	−0.3123 (2)	0.25359 (6)	0.0473 (4)	
H7	0.7032	−0.4082	0.2226	0.057*	
C8	0.73726 (12)	−0.1148 (2)	0.26736 (6)	0.0401 (3)	
C9	0.69806 (11)	0.0239 (2)	0.31228 (5)	0.0344 (3)	
H9	0.7394	0.1584	0.3198	0.041*	
C10	0.59639 (11)	−0.0312 (2)	0.34765 (5)	0.0325 (3)	
C11	0.62050 (10)	0.2897 (2)	0.42351 (5)	0.0316 (3)	
C12	0.75391 (10)	0.3095 (2)	0.42076 (5)	0.0297 (3)	
C13	0.82996 (11)	0.1359 (2)	0.43596 (5)	0.0340 (3)	
H13	0.7978	−0.0045	0.4462	0.041*	
C14	0.95292 (11)	0.1683 (2)	0.43615 (6)	0.0385 (3)	
H14	1.0042	0.0491	0.4468	0.046*	
C15	1.00296 (11)	0.3713 (2)	0.42111 (6)	0.0386 (3)	
C16	0.92554 (12)	0.5426 (2)	0.40584 (6)	0.0399 (3)	
H16	0.9577	0.6822	0.3949	0.048*	
C17	0.80264 (11)	0.5137 (2)	0.40630 (6)	0.0349 (3)	
H17	0.7513	0.6341	0.3967	0.042*	
C18	0.90578 (16)	0.1156 (3)	0.24644 (8)	0.0635 (5)	
H18A	0.8577	0.2483	0.2389	0.095*	
H18B	0.9770	0.1183	0.2206	0.095*	
H18C	0.9304	0.1118	0.2892	0.095*	
C19	1.13644 (12)	0.4059 (3)	0.42216 (7)	0.0555 (4)	
H19A	1.1608	0.4843	0.3853	0.083*	
H19B	1.1769	0.2623	0.4240	0.083*	
H19C	1.1586	0.4935	0.4579	0.083*	

### Atomic displacement parameters (Å^2^)

**Table:**

	*U*^11^	*U*^22^	*U*^33^	*U*^12^	*U*^13^	*U*^23^
O1	0.0303 (5)	0.0623 (7)	0.0510 (6)	0.0003 (4)	0.0060 (4)	0.0009 (5)
O2	0.0604 (6)	0.0584 (7)	0.0431 (5)	0.0026 (5)	0.0108 (5)	−0.0121 (5)
O3	0.0406 (5)	0.0471 (6)	0.0514 (6)	0.0026 (4)	0.0110 (4)	−0.0114 (5)
C1	0.0291 (6)	0.0354 (7)	0.0327 (6)	0.0000 (5)	−0.0054 (5)	0.0051 (5)
C2	0.0296 (6)	0.0481 (8)	0.0396 (7)	−0.0002 (6)	−0.0050 (5)	0.0082 (6)
C3	0.0324 (7)	0.0561 (9)	0.0541 (8)	−0.0112 (6)	−0.0057 (6)	0.0105 (7)
C4	0.0416 (7)	0.0441 (8)	0.0560 (8)	−0.0145 (6)	−0.0166 (6)	0.0096 (7)
C5	0.0435 (7)	0.0334 (7)	0.0398 (7)	−0.0028 (6)	−0.0157 (5)	0.0046 (5)
C6	0.0557 (9)	0.0342 (7)	0.0466 (8)	−0.0024 (6)	−0.0201 (6)	−0.0019 (6)
C7	0.0633 (9)	0.0395 (8)	0.0389 (7)	0.0102 (7)	−0.0136 (6)	−0.0106 (6)
C8	0.0470 (7)	0.0423 (8)	0.0309 (6)	0.0055 (6)	−0.0032 (5)	−0.0022 (5)
C9	0.0398 (7)	0.0318 (6)	0.0314 (6)	−0.0002 (5)	−0.0057 (5)	−0.0008 (5)
C10	0.0333 (6)	0.0318 (6)	0.0322 (6)	0.0004 (5)	−0.0096 (5)	0.0043 (5)
C11	0.0321 (6)	0.0348 (6)	0.0281 (6)	0.0017 (5)	0.0015 (4)	0.0013 (5)
C12	0.0318 (6)	0.0309 (6)	0.0263 (5)	−0.0006 (5)	−0.0018 (4)	−0.0041 (5)
C13	0.0373 (6)	0.0297 (6)	0.0350 (6)	−0.0010 (5)	−0.0019 (5)	0.0011 (5)
C14	0.0364 (7)	0.0419 (7)	0.0371 (6)	0.0080 (6)	−0.0061 (5)	0.0001 (5)
C15	0.0331 (6)	0.0498 (8)	0.0330 (6)	−0.0039 (6)	−0.0020 (5)	−0.0047 (6)
C16	0.0407 (7)	0.0354 (7)	0.0436 (7)	−0.0088 (6)	−0.0003 (5)	−0.0008 (6)
C17	0.0377 (7)	0.0293 (6)	0.0377 (6)	0.0008 (5)	−0.0033 (5)	−0.0015 (5)
C18	0.0617 (10)	0.0691 (11)	0.0600 (10)	−0.0069 (9)	0.0244 (8)	−0.0082 (8)
C19	0.0334 (7)	0.0789 (12)	0.0542 (9)	−0.0074 (7)	−0.0031 (6)	−0.0031 (8)

### Geometric parameters (Å, °)

**Table:**

O1—C2	1.3433 (17)	C9—C10	1.4195 (18)
O1—H1	0.953 (15)	C9—H9	0.9500
O2—C8	1.3670 (17)	C11—C12	1.4953 (16)
O2—C18	1.424 (2)	C12—C17	1.3885 (18)
O3—C11	1.2332 (15)	C12—C13	1.3907 (17)
C1—C2	1.4031 (17)	C13—C14	1.3862 (18)
C1—C10	1.4414 (18)	C13—H13	0.9500
C1—C11	1.4704 (17)	C14—C15	1.390 (2)
C2—C3	1.410 (2)	C14—H14	0.9500
C3—C4	1.349 (2)	C15—C16	1.390 (2)
C3—H3	0.9500	C15—C19	1.5043 (18)
C4—C5	1.420 (2)	C16—C17	1.3827 (18)
C4—H4	0.9500	C16—H16	0.9500
C5—C6	1.411 (2)	C17—H17	0.9500
C5—C10	1.4237 (18)	C18—H18A	0.9800
C6—C7	1.354 (2)	C18—H18B	0.9800
C6—H6	0.9500	C18—H18C	0.9800
C7—C8	1.415 (2)	C19—H19A	0.9800
C7—H7	0.9500	C19—H19B	0.9800
C8—C9	1.3708 (18)	C19—H19C	0.9800
			
C2—O1—H1	105.1 (11)	O3—C11—C12	116.30 (11)
C8—O2—C18	117.68 (11)	C1—C11—C12	123.23 (11)
C2—C1—C10	118.60 (12)	C17—C12—C13	119.27 (11)
C2—C1—C11	117.02 (11)	C17—C12—C11	118.17 (11)
C10—C1—C11	124.33 (11)	C13—C12—C11	122.42 (11)
O1—C2—C1	124.04 (12)	C14—C13—C12	119.75 (12)
O1—C2—C3	114.79 (12)	C14—C13—H13	120.1
C1—C2—C3	121.11 (13)	C12—C13—H13	120.1
C4—C3—C2	120.21 (13)	C13—C14—C15	121.57 (12)
C4—C3—H3	119.9	C13—C14—H14	119.2
C2—C3—H3	119.9	C15—C14—H14	119.2
C3—C4—C5	121.68 (13)	C16—C15—C14	117.84 (12)
C3—C4—H4	119.2	C16—C15—C19	120.94 (13)
C5—C4—H4	119.2	C14—C15—C19	121.22 (13)
C6—C5—C4	121.28 (13)	C17—C16—C15	121.28 (12)
C6—C5—C10	119.50 (13)	C17—C16—H16	119.4
C4—C5—C10	119.21 (13)	C15—C16—H16	119.4
C7—C6—C5	121.63 (13)	C16—C17—C12	120.26 (12)
C7—C6—H6	119.2	C16—C17—H17	119.9
C5—C6—H6	119.2	C12—C17—H17	119.9
C6—C7—C8	119.29 (13)	O2—C18—H18A	109.5
C6—C7—H7	120.4	O2—C18—H18B	109.5
C8—C7—H7	120.4	H18A—C18—H18B	109.5
O2—C8—C9	123.90 (13)	O2—C18—H18C	109.5
O2—C8—C7	115.18 (12)	H18A—C18—H18C	109.5
C9—C8—C7	120.92 (13)	H18B—C18—H18C	109.5
C8—C9—C10	120.74 (12)	C15—C19—H19A	109.5
C8—C9—H9	119.6	C15—C19—H19B	109.5
C10—C9—H9	119.6	H19A—C19—H19B	109.5
C9—C10—C5	117.76 (12)	C15—C19—H19C	109.5
C9—C10—C1	123.31 (11)	H19A—C19—H19C	109.5
C5—C10—C1	118.90 (12)	H19B—C19—H19C	109.5
O3—C11—C1	120.38 (11)		

### Hydrogen-bond geometry (Å, °)

**Table:**

CT1 and CT2 are the centroids of the C5–C10 and C12-C17 rings, respectively.

**Table:**

*D*—H···*A*	*D*—H	H···*A*	*D*···*A*	*D*—H···*A*
O1—H1···O3	0.95 (2)	1.70 (2)	2.5618 (14)	148 (2)
O1—H1···O3^i^	0.95 (2)	2.33 (2)	3.0083 (16)	128 (1)
C6—H6···CT1^ii^	0.95	2.71	3.5203 (13)	144
C17—H17···CT1^iii^	0.95	2.76	3.5492 (12)	141
C19—H19C···CT2^iv^	0.98	2.88	3.7834 (16)	154

Symmetry codes: (i) −*x*+1, −*y*+1, −*z*+1; (ii) −*x*+1, *y*−1/2, −*z*+1/2; (iii) *x*, *y*+1, *z*; (iv) −*x*+2, −*y*+1, −*z*+1.
